# From Multiplex Serology to Serolomics—A Novel Approach to the Antibody Response against the SARS-CoV-2 Proteome

**DOI:** 10.3390/v13050749

**Published:** 2021-04-24

**Authors:** Julia Butt, Rajagopal Murugan, Theresa Hippchen, Sylvia Olberg, Monique van Straaten, Hedda Wardemann, Erec Stebbins, Hans-Georg Kräusslich, Ralf Bartenschlager, Hermann Brenner, Vibor Laketa, Ben Schöttker, Barbara Müller, Uta Merle, Tim Waterboer

**Affiliations:** 1 Infections and Cancer Epidemiology, German Cancer Research Center (Deutsches Krebsforschungszentrum, DKFZ), 69120 Heidelberg, Germany; j.butt@dkfz.de; 2 B Cell Immunology, German Cancer Research Center (Deutsches Krebsforschungszentrum, DKFZ), 69120 Heidelberg, Germany; r.murugan@dkfz.de (R.M.); h.wardemann@dkfz.de (H.W.); 3 Department of Internal Medicine IV, University Hospital Heidelberg, 69120 Heidelberg, Germany; Theresa.Hippchen@med.uni-heidelberg.de (T.H.); uta.merle@med.uni-heidelberg.de (U.M.); 4 Department of Infectious Diseases, Virology, University Hospital Heidelberg, 69120 Heidelberg, Germany; Sylvia.Olberg@uni-heidelberg.de (S.O.); hans-georg.kraeusslich@med.uni-heidelberg.de (H.-G.K.); vibor.laketa@uni-heidelberg.de (V.L.); Barbara.Mueller@med.uni-heidelberg.de (B.M.); 5 German Center for Infection Research (DZIF), Heidelberg Partner Site, 69120 Heidelberg, Germany; r.bartenschlager@dkfz.de; 6 Division of Structural Biology of Infection and Immunity, German Cancer Research Center (Deutsches Krebsforschungszentrum, DKFZ), 69120 Heidelberg, Germany; m.vanstraaten@dkfz-heidelberg.de (M.v.S.); e.stebbins@dkfz.de (E.S.); 7 Department of Infectious Diseases, Molecular Virology, Heidelberg University, 69120 Heidelberg, Germany; 8 Division Virus-Associated Carcinogenesis, German Cancer Research Center (DKFZ), 69120 Heidelberg, Germany; 9 Division of Clinical Epidemiology and Aging Research, German Cancer Research Center (Deutsches Krebsforschungszentrum, DKFZ), 69120 Heidelberg, Germany; h.brenner@dkfz.de (H.B.); b.schoettker@dkfz.de (B.S.); 10 Network Aging Research, University of Heidelberg, 69120 Heidelberg, Germany

**Keywords:** SARS-CoV-2, multiplex serology

## Abstract

The emerging SARS-CoV-2 pandemic entails an urgent need for specific and sensitive high-throughput serological assays to assess SARS-CoV-2 epidemiology. We, therefore, aimed at developing a fluorescent-bead based SARS-CoV-2 multiplex serology assay for detection of antibody responses to the SARS-CoV-2 proteome. Proteins of the SARS-CoV-2 proteome and protein N of SARS-CoV-1 and common cold Coronaviruses (ccCoVs) were recombinantly expressed in *E. coli* or HEK293 cells. Assay performance was assessed in a COVID-19 case cohort (*n* = 48 hospitalized patients from Heidelberg) as well as *n* = 85 age- and sex-matched pre-pandemic controls from the ESTHER study. Assay validation included comparison with home-made immunofluorescence and commercial enzyme-linked immunosorbent (ELISA) assays. A sensitivity of 100% (95% CI: 86–100%) was achieved in COVID-19 patients 14 days post symptom onset with dual sero-positivity to SARS-CoV-2 N and the receptor-binding domain of the spike protein. The specificity obtained with this algorithm was 100% (95% CI: 96–100%). Antibody responses to ccCoVs N were abundantly high and did not correlate with those to SARS-CoV-2 N. Inclusion of additional SARS-CoV-2 proteins as well as separate assessment of immunoglobulin (Ig) classes M, A, and G allowed for explorative analyses regarding disease progression and course of antibody response. This newly developed SARS-CoV-2 multiplex serology assay achieved high sensitivity and specificity to determine SARS-CoV-2 sero-positivity. Its high throughput ability allows epidemiologic SARS-CoV-2 research in large population-based studies. Inclusion of additional pathogens into the panel as well as separate assessment of Ig isotypes will furthermore allow addressing research questions beyond SARS-CoV-2 sero-prevalence.

## 1. Introduction

The SARS-CoV-2 pandemic has emerged worldwide, but there is still a lack of knowledge on the epidemiology of infection. Large-scale population-based studies would not only provide reliable prevalence estimates but also identify factors associated with the infection and transmission. Consequently, there is an urgent need for assays that provide high-throughput methodology. Direct detection of the infectious SARS-CoV-2 or its RNA genome is limited to a specific time frame after infection and only provides information about current but not past infections. In contrast, antibody responses indicate current and past infections and allow for a cross-sectional assessment of SARS-CoV-2 cumulative exposure in a given population. Current serological assays are mostly ELISA- or (electro)chemiluminescence-based and limited to a single antigen, either the nucleocapsid protein (N) or the spike protein (S) [1]. Often, subdomains of S are used as antigens, including the N-terminal S1 domain, which is cleaved from the C-terminal S2 domain during host cell attachment and entry, and the receptor-binding domain (RBD) as part of the S1 domain [1]. Both, S1 and S1-RBD are highly glycosylated, which is important for correct conformation of the protein [2]. Proteins N and S share high sequence homologies to their counterparts of other Coronaviruses (CoVs), including SARS-CoV-1, and endemic common cold CoVs (ccCoVs) NL63, 229E, HKU1, and OC43, potentially resulting in cross-reactive antibody responses and consequently lowered specificity [3]. A multiplex approach analyzing multiple antigens in a time- and labor-efficient manner would potentially increase specificity of detecting current and/or past SARS-CoV-2 exposure, and allow identifying antibody patterns meaningful for, e.g., prediction of disease course.

Thus far, only a few studies have employed either microarray or fluorescent-bead based technologies to develop multiplex SARS-CoV-2 serological assays [4,5,6,7,8,9], all providing high specificity and sensitivity in detecting SARS-CoV-2 antibodies by varying combinations of proteins N and S, as well as subdomains or peptides thereof. Microarray-based studies utilized peptides or proteins of the SARS-CoV-2 proteome [5,6,7] allowing for assessment of the immunogenicity of proteins other than N and S. In contrast to fluorescent-bead based technologies, microarray-based assays are, however, not suited for high-throughput analyses of large sample sets.

Here, we report the development of a fluorescent-bead based SARS-CoV-2 multiplex serology assay for the detection of antibody responses to the SARS-CoV-2 proteome, including proteins N and S, either in full-length or as their respective subdomains N-EP3 (a predicted B-cell epitope of protein N [10]) and S1, S1-RBD, S2, and a shorter fragment S2′ [11,12]. This set-up will potentially allow achieving an exceptionally high specificity and sensitivity by combined antigen algorithms for SARS-CoV-2 sero-positivity. In addition, we aimed to include proteins of the entire SARS-CoV-2 proteome to allow for association studies beyond mere sero-prevalence, as well as the N proteins of related CoVs to assess potential cross-reactive antibody responses. We furthermore aimed for performing assay validation against multiple gold-standard assays in a well-characterized local case cohort of COVID-19 patients as well as age- and sex-matched pre-pandemic controls.

## 2. Methods

### 2.1. Study Population

The cohort of COVID-19 cases included in this study was recruited at the Heidelberg University Clinics (Heidelberg, Germany) between 18th March and 22nd May 2020. Presence of SARS-CoV-2 infection was confirmed by RT-PCR as described previously [13]. Briefly, RNA was isolated from nasopharyngeal swabs using QIAGEN Kits (QIAGEN, Hilden, Germany) automated on the QIAsymphony instrument (DSP Virus pathogen mini kits). Extracted samples were used in a RT-PCR reaction, carried out using various reagent mixes: LightMix Modular SARS and Wuhan CoV E-gene, LightMix Modular SARS and Wuhan CoV N-gene, LightMix Modular Wuhan CoV RdRP-gene and LightMix Modular EAV RNA Extraction Control (TIBMOLBIOL, Berlin, Germany), and LightCycler Multiplex RNA Virus Master (Roche, Mannheim, Germany) according to manufacturer’s instructions. RT-PCR was performed on LightCycler 480 instruments (Roche, Mannheim, Germany). Tests were performed and interpreted according to the manufacturer’s instructions. Participants provided information on age, sex, and COVID-19 symptoms, if any, as well as date of symptom onset. The need and type of oxygenation as well as death due to COVID-19 in hospitalized patients were documented. Blood samples were drawn from each participant at baseline and in the follow-up in varying time intervals dependent on whether the patient was hospitalized and the duration of hospital stay. In total, *n* = 192 serum samples were shipped to the German Cancer Research Center (DKFZ, Heidelberg, Germany) for serological analyses. Of these, *n* = 18 samples had to be excluded due to mis-labelling of the sample tube, technical errors that had occurred during the multiplex serology analysis, a negative SARS-CoV-2 PCR test, or missing information on onset of symptoms. Thus, the final serum set included in total *n* = 174 sera. *n* = 156 serum samples originated from *n* = 48 hospitalized COVID-19 patients, and *n* = 18 serum samples originated from *n* = 15 COVID-19 patients who experienced only mild symptoms and were not hospitalized. The median age among hospitalized patients was 62 years (range: 23–85 years) and 33% of hospitalized patients were female. Among non-hospitalized patients, the median age was 54 years (range: 27–70 years) and 60% were female.

The study was approved by the Ethics Committee of the Medical Faculty Heidelberg (approval number S-148/2020, date of approval March 18, 2020) and conducted in accordance with the Declaration of Helsinki.

Serum samples from pre-pandemic controls were obtained from the ESTHER I study (“Epidemiologische Studie zu Chancen der Verhütung, Früherkennung und optimierten Therapie chronischer Erkrankungen in der älteren Bevölkerung”), a prospective cohort that enrolled in total of 9940 subjects aged 50–75 years between July 2000 and December 2002 in Saarland, Germany. Participants were recruited by their general practitioner during a general health check-up examination who provided a blood sample as well as sociodemographic information [14]. We randomly selected *n* = 88 participants to frequency match the age- and sex-distribution among the above-described hospitalized *n* = 48 COVID-19 patients. After multiplex serology analysis, *n* = 3 serum samples were excluded due to technical errors. The median age among the remaining *n* = 85 pre-pandemic controls was 62 years (range: 50–75 years) and 33% were female.

The ESTHER study was approved by the ethics committees of the University of Heidelberg (approval number S-058/2000) and the medical board of the state of Saarland (approval number 67/00). Written informed consent was obtained from each participant.

### 2.2. Selection and Recombinant Expression of SARS-CoV-2 Proteins

For the development of SARS-CoV-2 multiplex serology, we attempted to express the near-complete SARS-CoV-2 proteome (NCBI accession no. NC_045512.2) (Table 1) [15]. Non-structural proteins (NSP) 3, 4, and 6 were not included in the antigen panel, since these proteins were predicted to have multiple hydrophobic regions posing challenges to efficient expression. We furthermore excluded NSP11 because this protein is composed of only 11 amino acids. Since the SARS-CoV-2 spike (S) protein is cleaved by host furin, we included the respective fragments S1 and S2, as well as an additional shorter fragment of S2, i.e., S2′ [12]. Proteins N and S1 were previously used in serological analyses [1]. We additionally included SARS-CoV-2 sub-fragments (N-EP3 and S1-RBD) that might induce less cross-reactive antibody responses to related Coronaviruses [10]. All proteins were recombinantly expressed as fusion proteins in *E. coli* BL21 with an N-terminal glutathione S-transferase (GST) and a C-terminal tag consisting of the 11 C-terminal amino acid residues of the large T antigen of simian virus 40, as described previously [16]. Gene synthesis and subcloning in the respective vector pGEX4T3tag were performed by Eurofins (Ebersberg, Germany). All recombinantly expressed proteins underwent quality controls to confirm the correctness of the construct (DNA extraction and PCR with subsequent sequence analysis) as well as sufficient expression yields of full-length protein (semi-quantitative ELISA to detect the C-terminal tag sequence and Western blots against both terminal tags). All proteins but NSP12 and NSP13 passed the quality controls. These two proteins were therefore excluded from further analyses.

Since protein S1 and the sub-fragment S1-RBD were reported to be highly glycosylated [2], which might be of importance for the immunogenicity of the proteins, we included both proteins also as purified his-tagged proteins recombinantly expressed in eukaryotic HEK293 cells. S1-RBD was expressed as described previously [11], and S1 was purchased from Sino Biological (Eschborn, Germany). Indeed, bacterially expressed proteins S1 and S1-RBD showed little immunogenicity in comparison to their counterparts expressed in HEK293 cells (Supplementary Figure S1A). Therefore, only his-tagged S1 and S1-RBD expressed in HEK293 cells were considered for further analysis.

Antibody responses to SARS-CoV-2 protein N are speculated to potentially result from cross-reactive responses after infection with common cold Coronaviruses (ccCoVs) [3]. To assess potential cross-reactivity in the measured antibody responses, we included protein N of SARS-1 (NCBI accession no. NP_828858.1), and ccCoVs NL63 (NCBI accession no. YP_003771.1), 229E (NCBI accession no. NP_073556.1), OC43 (NCBI accession no. AAT84358.1), and HKU1 (NCBI accession no. YP_173242.1) (Table 1). These proteins were recombinantly expressed as GST-tagged fusion proteins in *E. coli* BL21 as described above.

### 2.3. Multiplex Serology

The GST-tagged fusion proteins were affinity-purified on glutathione-casein coated fluorescently labelled polystyrene beads (Luminex Corp., Austin, TX, USA), whereas purified his-tagged proteins were directly cross-linked to the bead surface, as described previously [16,17].

Mixing the bead sets loaded with distinct antigens allowed a high-throughput simultaneous analysis of several antigens per serum. For all secondary antibodies, sera were pre-incubated (1:50 dilution) in a buffer containing polyvinyl alcohol, polyvinyl pyrrolidone, casein, and protein lysate of *E. coli* over-expressing GST-tag to suppress unspecific binding of antibodies to the glutathione-coated beads and residual *E. coli* proteins. For simultaneous detection of IgM/IgA/IgG, sera were additionally pre-incubated with 2.5% Super ChemiBlock™ Heterophile Blocking Agent (CBS-K, Chemicon, Temecula, CA, USA) [18], and for detection of IgM, sere were pre-incubated with 1:10 diluted Rf-absorbens (Linaris, Mannheim, Germany). After the pre-incubation step, sera were mixed (1:1) and incubated with the antigen-loaded bead mixture. Bound serum antibodies were labelled separately with biotinylated secondary antibodies (goat anti-human IgM/IgA/IgG, anti-human IgM, anti-human IgG, or anti-human IgA; Jackson ImmunoResearch, West Grove, PA, USA) and subsequently incubated with Streptavidin-R-Phycoerythrin (MossBio, Pasadena, MD, USA). Simultaneous detection of IgM/IgA/IgG is reported unless otherwise specified. A Luminex 200 Analyzer (Luminex Corp., Austin, TX, USA) was used to distinguish the bead sets and their respective antigens and to quantify the amount of bound serum antibody. The level of antibody response is given as median fluorescence intensity (MFI) of at least 100 beads per type measured. Background values against the GST-tag, as well as the bead-surface and secondary reagents were subtracted to generate net MFI values.

Antigen-specific cut-offs for sero-positivity (Table 1) were defined as the mean plus three times the standard deviation among the analyzed population sampled before the COVID-19 pandemic (pre-pandemic controls). The technical minimum cut-off was 100 MFI, except for Protein S1 expressed in HEK293 cells (1000 MFI) due to observed variations in background values.

### 2.4. SARS-CoV-2 IgG ELISA

For *n* = 124 of the total of *n* = 241 serum samples analyzed in multiplex serology, we performed a concurrent SARS-CoV-2 IgG enzyme-linked immunosorbent assay (ELISA) specific for antibodies directed against the S1 domain of the spike protein (Euroimmun, Lübeck, Germany). The samples were analyzed on a Euroimmun Analyzer I instrument according to the manufacturer’s instructions. Briefly, serum was applied in a 1:101 dilution on the provided antigen-coated ELISA plates, and subsequently incubated with a peroxidase-linked anti-human IgG secondary antibody. The amount of bound serum antibody was measured as the optical density (OD) at 450 nm after incubation with substrate solution. The ratio of the obtained OD per sample and the extinction of a calibrator sample were calculated. Following the manufacturer’s protocol, a ratio < 0.8 was considered sero-negative, a ratio ≥ 1.1 was considered sero-positive, and ratios between 0.8 and 1.0 were considered borderline.

### 2.5. Microscopy-Based Immunofluorescence Detection of IgG Antibodies to SARS-CoV-2

Microscopy-based immunofluorescence detection of IgG antibodies to SARS-CoV-2 antibodies was performed using a newly established semi-automated, semi-quantitative approach, as described previously [19], for *n* = 38 serum samples of hospitalized COVID-19 patients. In contrast to the ELISA measurements, this approach is not specific for a single viral protein, but can detect antibodies against all viral proteins expressed in the infected cell context. Briefly, fixed and permeabilized SARS-CoV-2-infected VeroE6 cells in 96-well plates were incubated with patient serum or control serum, respectively. Bound serum IgG antibodies to SARS-CoV-2 were detected using goat anti-human IgG-Alexa Fluor 488 (Invitrogen, Thermo Fisher Scientific) and evaluated by fluorescence microscopy. Concomitant immunostaining against dsRNA was performed in order to differentiate between virus-expressing cells (dsRNA positive) and non-infected cells (dsRNA negative) in the same specimen. Semi-automated microscopy and image data analysis was performed as described in detail in another study [19]. For each sample, a score was calculated based on the ratio of immunostaining intensity determined for cells infected with SARS-CoV-2 to that of the non-infected cells in the same specimen. A threshold value of 1.30 for sero-positivity was defined [19] based on receiver operating characteristic (ROC) analysis to achieve optimum specificity. Scores < 1.27 were considered sero-negative, and those between 1.27 and 1.30 as borderline to allow for better sensitivity of the assay [19].

### 2.6. Statistical Analysis

Continuous antibody responses to SARS-CoV-2 proteins were compared between pre-pandemic controls and hospitalized COVID-19 patients using the Wilcoxon rank sum test. A *p*-value < 0.05 was considered statistically significant. Agreement between SARS-CoV-2 multiplex serology and commercial SARS-CoV-2 ELISA (Euroimmun, Lübeck, Germany) was assessed using Cohen’s kappa and the respective 95% confidence interval (CI).

All graphical and descriptive representations were performed using SAS 9.4 (SAS Institute, Cary, NC, USA) or GraphPad Prism 8 (GraphPad Software, Inc., San Diego, CA, USA).

All summary data generated or analyzed during this study are included in this published article and its Supplementary Material file. The primary data are available from the corresponding author on reasonable request.

## 3. Results

### 3.1. Antibody Responses to SARS-CoV-2 Proteins in Pre-Pandemic Controls and Hospitalized COVID-19 Patients

**Figure 1:**
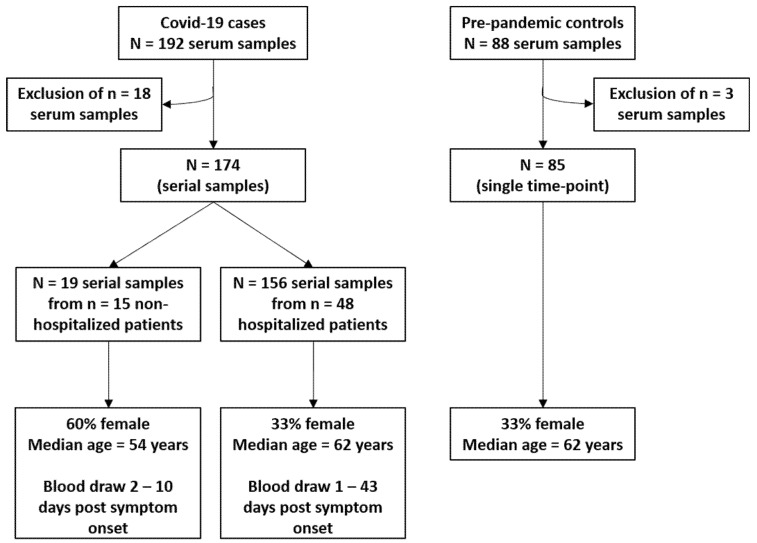
Schematic study diagram. COVID-19 cases were recruited at Heidelberg University Clinics between March and May 2020. Of the *n* = 192 serum samples, *n* = 18 samples were excluded due to technical errors, a negative SARS-CoV-2 PCR test, or missing information about onset of symptoms. Pre-pandemic controls were obtained from the ESTHER I study (“Epidemiologische Studie zu Chancen der Verhütung, Früherkennung und optimierten Therapie chronischer Erkrankungen in der älteren Bevölkerung”), a prospective cohort that enrolled subjects aged 50–75 years between 2000 and 2002 in Saarland, Germany. We randomly selected *n* = 88 participants to frequency match the age- and sex-distribution among the hospitalized *n* = 48 COVID-19 patients. Due to technical errors, *n* = 3 serum samples were excluded.

**Figure 2:**
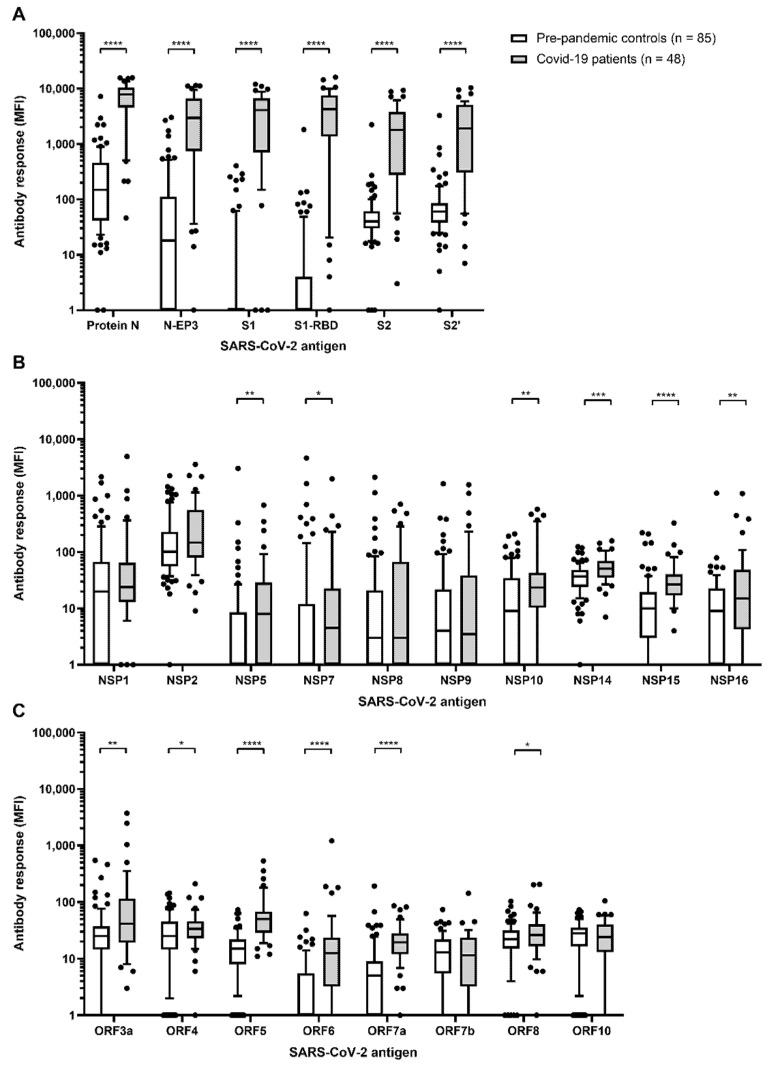
Antibody responses to SARS-CoV-2 proteins in *n* = 85 pre-pandemic controls and *n* = 48 hospitalized COVID-19 patients. (**A**) Antibody responses (median fluorescence intensity (MFI)) to nucleocapsid (N) and spike (S) proteins, as well as their respective fragments; (**B**) SARS-CoV-2 non-structural proteins (NSP); (**C**) other SARS-CoV-2 open reading frames (ORF). If multiple samples per individual were available only the sample with the latest time point after blood draw was considered for analysis. Boxes represent the 25th to 75th and whiskers the 10th to 90th percentiles, respectively. Wilcoxon rank sum test was applied to assess statistically significant differences in antibody responses (MFI) to SARS-CoV-2 proteins between pre-pandemic controls and hospitalized COVID-19 patients; * *p*-value < 0.05, ** *p*-value < 0.01, *** *p*-value < 0.001, **** *p*-value < 0.0001.

An overview of the study design is shown in Figure 1. Antibody responses to all proteins included in SARS-CoV-2 multiplex serology were compared between *n* = 85 pre-pandemic controls and *n* = 48 hospitalized COVID-19 patients based on a single serum sample drawn at the latest time point after symptom onset (range: 2–43 days) (Figure 2).

Among controls, protein N exerted the highest antibody response of all proteins analyzed (median: 149 MFI, range: 1–7195 MFI) (Figure 2A). This was also reflected in the comparably high cut-off of 3133 MFI determined as the mean plus three times the standard deviation among pre-pandemic controls (Table 1). For all other proteins, the median antibody response ranged between 1 and 101 MFI, and the cut-off values were much lower.

We further assessed whether the observed high responses to protein N result from cross-reactive antibody responses potentially originating from previous infections with SARS-CoV-1 and endemic ccCoVs NL63, HKU1, 229E, and OC43. Antibody responses to proteins N of OC43, HKU1, NL63, and 229E were abundantly high in pre-pandemic controls (>1000 MFI) and consequently, there was little correlation with antibody responses to protein N of SARS-CoV-2 (Supplementary Figure S2B,C). However, antibody responses to the N proteins of SARS-CoV-1 and SARS-CoV-2 were correlated (R^2^ = 0.55) (Supplementary Figure S2A).

Proteins with the highest levels of immunogenicity in COVID-19 patients were proteins N, S1, and S2 (median: 7825, 4098, and 1789 MFI, respectively) as well as their respective sub-fragments N-EP3, S1-RBD, and S2′ (median: 2943, 4228, and 1030 MFI, respectively) (Figure 2A). Antibody responses to these proteins were all significantly higher among COVID-19 patients compared to pre-pandemic controls (all *p*-values < 0.0001, Figure 2A). For all other proteins included in the multiplex serology assay, except NSP1, NSP2, NSP8, NSP9, ORF7a, and ORF10, antibody responses were also significantly higher among cases than controls (all *p*-values < 0.05). However, overall antibody responses to these proteins were comparably low among cases with medians ranging between 4 and 147 MFI, and mostly below the determined cut-off values (Table 1).

**Figure 3:**
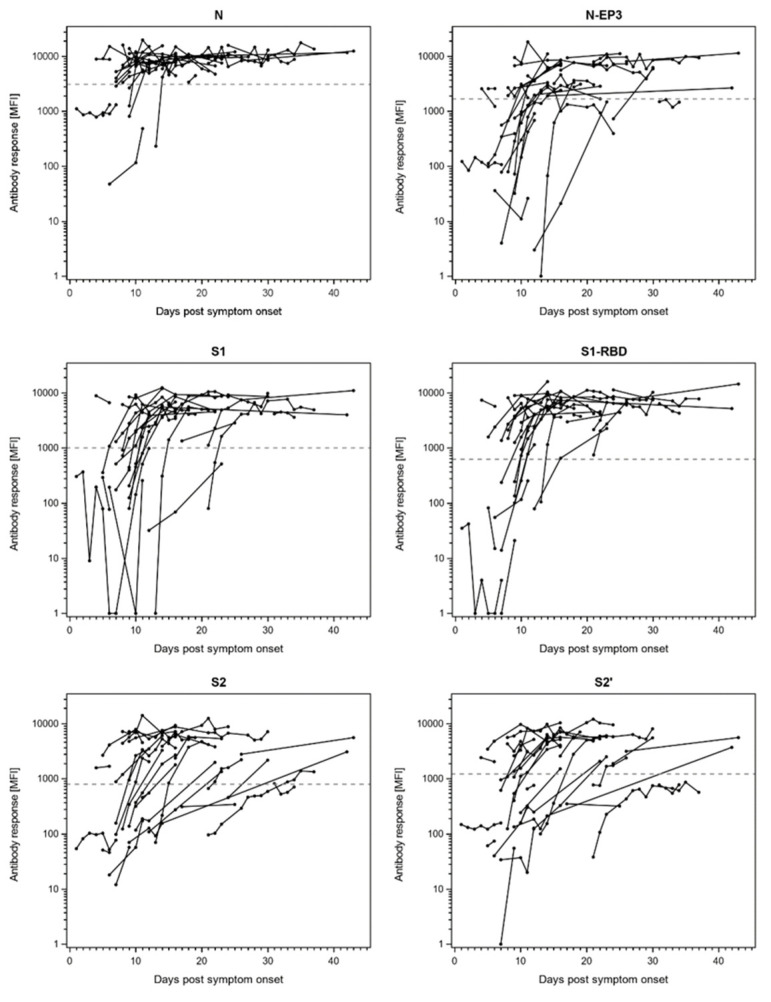
Antibody responses to SARS-CoV-2 proteins N, S1, S2, and their respective sub-fragments (N-EP3, S1-receptor-binding domain (RBD), and S2′) in longitudinal samples of *n* = 32 hospitalized COVID-19 patients. The antibody response (MFI) is plotted against days post symptom onset. The dashed lines indicate the antigen-specific cut-offs defined as mean plus three times the standard deviation in *n* = 85 pre-pandemic controls.

Since the COVID-19 patients in our cohort were sampled at various time points after symptom onset, we analyzed the available longitudinal samples for sero-conversion (Figure 3 and Supplementary Figure S3). Indeed, for the most immunogenic proteins N, N-EP3, S1, S1-RBD, S2, and S2′ the majority of hospitalized COVID-19 patients sero-converted at the latest 2 weeks after symptom onset (Figure 3). For all other proteins, sero-conversion was a rare event and was only observed for individual patients at distinct time-points after symptom onset (Supplementary Figure S3).

### 3.2. Assessment of Sensitivity and Specificity of the Newly Developed SARS-CoV-2 Multiplex Serology Assay

Based on the specific timing of sero-conversion to SARS-CoV-2 proteins described above, we assessed sensitivity of the newly developed multiplex serology assay separately for hospitalized COVID-19 patients, who provided their latest blood sample ≤ 14 days (*n* = 23) or > 14 days (*n* = 25) after symptom onset (Table 2). Among patients with a blood sample drawn > 14 days after symptom onset, the highest sero-prevalence was achieved with proteins N and S1-RBD (both 100%, 95% CI: 86–100%), followed by protein S1 (96%, 95% CI: 80–99%), proteins S2 and N-EP3 (both 80%, 95% CI: 59–93%), and protein fragment S2′ (76%, 95% CI: 55–91%). Sero-prevalence for the other SARS-CoV-2 proteins was low and reached at most 16% (*n* = 4 sero-positive individuals) among hospitalized patients with a blood sample drawn > 14 days after symptom onset.

Antibody responses to S1 and S1-RBD strongly correlated with each other (R^2^ = 0.93) (Supplementary Figure S1B). Since S1-RBD showed a slightly higher prevalence in hospitalized patients with blood drawn ≤ 14 days after symptom onset (57%, 95% CI: 34–77%) than S1 (48%, 95% CI: 27–69%), we considered S1-RBD as parameter for a final algorithm defining SARS-CoV-2 sero-positivity (Table 2).

Protein N-EP3 showed substantially lower sensitivity than the full-length protein N (80% versus 100%, respectively); and proteins S2 and S2′ also only achieved sensitivities of up to 80% in hospitalized patients more than 2 weeks after symptom onset. Consequently, proteins N-EP3, S2, and S2′ do not further contribute to an increased sensitivity in addition to Protein N and S1-RBD. Defining SARS-CoV-2 overall sero-positivity as being dual-positive to Protein N and S1-RBD resulted in 100% sensitivity (95% CI: 86–100%) in detecting hospitalized COVID-19 patients >14 days after symptom onset. Among hospitalized patients with a shorter time between symptom onset and blood draw, the sensitivity of our assay was 48% (95% CI: 27–69%). Interestingly, among non-hospitalized patients (*n* = 15), all sampled within 14 days after symptom onset, only one individual (7%) was considered sero-positive with SARS-CoV-2 multiplex serology.

Among pre-pandemic controls, only one individual each was sero-positive to N or S1-RBD, respectively. However, none of the pre-pandemic controls was dual-positive and thus considered SARS-CoV-2 sero-positive, indicating 100% (95% CI: 96–100%) specificity of our newly developed assay.

### 3.3. Comparison of SARS-CoV-2 Multiplex Serology to a Commercially Available ELISA and Microscopy-Based Immunofluorescence

We generated concurrent data from a widely used commercial SARS-CoV-2 IgG ELISA (Euroimmun, Lübeck, Germany) for *n* = 124 serum samples (*n* = 30 pre-pandemic controls, *n* = 88 serum samples from *n* = 25 hospitalized COVID-19 patients, and *n* = 6 serum samples from *n* = 5 non-hospitalized COVID-19 patients). Two of the analyzed serum samples were classified as borderline in ELISA. These samples consisted of one pre-pandemic control, who was considered negative with SARS-CoV-2 multiplex serology (Protein N + S1-RBD sero-negative), and one COVID-19 patient, who was defined positive with SARS-CoV-2 multiplex serology.

The ELISA definition for being sero-negative or -positive for SARS-CoV-2 was in substantial agreement (Cohen’s kappa = 0.68; 95% CI: 0.56–0.80) with being dual-positive to protein N and S1-RBD in multiplex serology (Table 3). All ELISA sero-positives (*n* = 49) were also sero-positive in multiplex serology. However, *n* = 20 of the *n* = 73 ELISA sero-negatives were defined positive in multiplex serology. These 20 samples consisted of *n* = 19 samples from hospitalized COVID-19 patients and *n* = 1 sample from a non-hospitalized COVID-19 patient with mild symptoms.

For *n* = 38 samples of hospitalized COVID-19 patients, we were able to compare our results to microscopy-based immunofluorescence detection of IgG antibodies to SARS-CoV-2. Of these, *n* = 1 sample was considered borderline in the microscopy-based assay but sero-negative in multiplex serology. For the remaining *n* = 37 samples, the microscopy-based assay and multiplex serology were in almost perfect agreement (Cohen’s kappa = 0.93; 95% CI: 0.81–1.00). Only one sample had a discordant result between the two assays and was considered sero-positive in the microscopy-based assay but sero-negative in multiplex serology (Table 3).

### 3.4. Sero-Positivity to SARS-CoV-2 Proteins Other Than N and S and Severity of COVID-19 Disease

In addition to whether patients were hospitalized, further information was available on oxygenation requirements and disease outcome of hospitalized patients. In an attempt to identify serological markers for disease severity, we included almost the entire SARS-CoV-2 proteome in our multiplex serology. Due to the small sample size, it was impossible to generate robust age- and sex-adjusted regression models, i.e., we were not able to adequately control for differences in patient characteristics. Therefore, we present a purely descriptive analysis (Table 4). Overall, sero-positivity to SARS-CoV-2 proteins other than N, N-EP3, S1, S1-RBD, S2, and S2′ was a rare event and observed in *n* = 10 hospitalized patients (20%) and in none of the non-hospitalized patients. Although all patients sero-positive to ≥ 2 SARS-CoV-2 proteins other than N and S or their sub-fragments were hospitalized (*n* = 10), the majority of them had a more favorable course of disease. *n* = 9 of these 10 patients were among the *n* = 45 patients that needed oxygenation during their hospital stay, however, 8 out of these 9 needed only oxygenation through a nasal cannula, in contrast to High-Flow Nasal Oxygen (HFNO) or invasive oxygenation. Of note, the single patient who did not need any oxygenation was sero-positive to the maximum number of proteins observed in this study (*n* = 5). Furthermore, none of the hospitalized patients that were sero-positive to at least two of the additional proteins died due to COVID-19.

### 3.5. Separate Detection of Immunoglobulin M, A, and G Antibody Responses in COVID-19 Patients with Longitudinal Samples

Besides measuring Ig classes M, A, and G simultaneously, we also assessed the antibody response to SARS-CoV-2 proteins separately by Ig class (Supplementary Figure S4). We here show IgM, IgA, and IgG responses to proteins N and S1-RBD for the 23 hospitalized COVID-19 patients with at least 3 follow-up samples. Only a single patient showed an isolated IgM increase against S1-RBD in the absence of IgG and/or IgA antibodies against the same protein (patient 23). In contrast, IgM responses against both protein N and S1-RBD were observed at the same time as IgG and/or IgA antibodies in the vast majority of patients. As expected, several patients showed increasing levels of all Ig classes against either protein N and/or S1-RBD over time, especially when sampled early after symptom onset (e.g., patients 4, 11, and 12). In the follow-up, the antibody responses by Ig class appeared distinct for the two proteins. In case of protein N, the IgM, IgA, and IgG responses remained fairly stable over the course of follow-up for many patients. For S1-RBD, however, the IgM and IgA responses decreased over the course of follow-up for some patients (e.g., 1 and 2), whereas IgG responses remained stable or even increased.

## 4. Discussion

We here present the development of a multiplex serology assay for detection of antibody responses to SARS-CoV-2. Defining dual sero-positivity to the nucleocapsid protein N and the RBD domain of spike protein S for classification as a positive result obtained 100% sensitivity and specificity in hospitalized COVID-19 patients and pre-pandemic controls. The assay was furthermore in excellent agreement with other serological SARS-CoV-2 assays. Inclusion of additional SARS-CoV-2 proteins in the antigen panel as well as assessment of different Ig classes allowed for further explorative analyses with progression of disease and course of antibody response.

The current SARS-CoV-2 pandemic leads to an urgent need for high-throughput approaches to assess SARS-CoV-2 epidemiology. We developed a multiplex serology assay based on fluorescent-bead based technology that includes the well-described SARS-CoV-2 immunogenic proteins N and S1 as well as their respective subdomains N-EP3 and S1-RBD [1,10,11]. We found that dual sero-positivity to proteins N and S1-RBD resulted in 100% sensitivity and 100% specificity when applied in hospitalized COVID-19 patients sampled > 14 days after symptom onset and age- and sex-matched pre-pandemic controls, respectively. Classification of SARS-CoV-2 sero-positivity separately by protein N or S1-RBD would have resulted in false-positive classifications of each one pre-pandemic control and consequently a lower specificity, highlighting the benefit of simultaneously assessing multiple antigens. This is further supported by comparison of SARS-CoV-2 multiplex serology with commercially available ELISA, detecting antibody responses to protein S1 only. Comparison of the S1-RBD protein applied in multiplex serology to ELISA results in substantial agreement (Cohen’s kappa = 0.65; 95% CI: 0.53–0.77) between the two assays; very similar to the comparison based on N and S1-RBD dual sero-positivity, i.e., the definition of overall SARS-CoV-2 sero-positivity in multiplex serology. However, one pre-pandemic control being defined as borderline in ELISA was defined sero-positive with the S1-RBD measurement in multiplex serology (data not shown). With the dual-classification for sero-positivity to proteins N and S1-RBD, this pre-pandemic control was correctly identified as sero-negative.

With respect to specificity of the assay, the potential of cross-reactive antibody responses resulting from previous infections with other CoVs, including SARS-CoV-1 and endemic ccCoVs NL63, HKU1, 229E, and OC43, should be acknowledged [3,8]. We observed the highest antibody responses among pre-pandemic controls to the nucleocapsid protein N, which exerts a strong potential for cross-reactive antibody responses due to high sequence similarity between related CoVs [3]. We therefore expressed the respective N proteins of SARS-CoV-1, OC43, HKU1, NL63, and 229E to address this issue. Antibody responses to proteins N of OC43, HKU1, NL63, and 229E were abundantly high (above 1000 MFI for the majority of pre-pandemic controls) and consequently there was little correlation with antibody responses to protein N of SARS-CoV-2, which is in concordance with one previously published study [8]. We can therefore not exclude that high antibody responses to SARS-CoV-2 protein N among controls may result from a previous infection with other CoVs, the most likely candidates being the ccCoVs [20]. However, our data also shows that not all high antibody responses to protein N of other CoVs necessarily induce cross-reactive responses to SARS-CoV-2. Antibody responses to the N proteins of SARS-CoV-1 and SARS-CoV-2 correlated (R^2^ = 0.55) but we do not expect any previous SARS-CoV-1 patients among our control group, since these were sampled before the SARS-CoV-1 epidemic in 2002/2003. Moreover, the SARS-CoV-1 pandemic was mostly localized in South-East Asia and our samples (controls and cases) were sampled in Germany. Thus, antibody responses to protein N most likely do not originate from previous SARS-CoV-1 infections in our population, however, they should be considered when analyzing studies from the respective time window and geographical regions. Based on our preliminary observations presented here, it will be important to include also the homologous spike proteins into the multiplex assay to more comprehensively assess potential cross-reactive antibody responses and to validate the N and spike protein responses against the ccCoVs with suitable reference material, especially sera from patients shortly after PCR-confirmed infection with other CoVs.

The newly developed SARS-CoV-2 multiplex serology not only provided the potential to increase specificity of SARS-CoV-2 serology but also showed a high sensitivity in detection of SARS-CoV-2-infected individuals. In comparison to abovementioned commercially available ELISA, we detected *n* = 20 (16%) discordant samples that were sero-negative in ELISA but classified sero-positive in SARS-CoV-2 multiplex serology. Of note, in samples that were part of a longitudinal series in patients (for 11 of these 20 samples), multiplex serology was able to identify COVID-19 patients as sero-positive up to 8 days earlier post symptom onset than the ELISA, indicating a higher sensitivity of SARS-CoV-2 multiplex serology. This might be in part due to the choice of secondary antibody. The commercially available ELISA used in this study detects IgG antibody responses only, whereas our secondary antibody for classification of SARS-CoV-2 sero-positivity detects IgM/IgA/IgG simultaneously. We performed a separate analysis with SARS-CoV-2 multiplex serology focusing on IgG and found both tests also to be in substantial agreement (Cohen’s kappa = 0.77; 95% CI: 0.66–0.88). However, for the IgG measurement, only *n* = 14 (versus *n* = 20 with simultaneous IgM/IgA/IgG detection) of the hospitalized COVID-19 patients were defined ELISA sero-negative but multiplex serology sero-positive. Similarly, the microscopy-based immunofluorescence detection of IgG antibodies to SARS-CoV-2 in comparison to the IgG measurement in multiplex serology showed two hospitalized COVID-19 patients being sero-negative in IgG multiplex serology but sero-positive in immunofluorescence, as opposed to only one discordant patient with the IgM/IgA/IgG measurement. Thus, application of a secondary antibody that detects multiple Ig isotypes simultaneously potentially allows for detection of individuals that had undergone SARS-CoV-2 infection that otherwise might have been missed, as indicated in the literature [21]. Taken together, our assay shows both 100% sensitivity and specificity, although these estimates come with relatively wide confidence intervals due to the limited size of our study. Replication in larger studies will be needed to confirm these results and to obtain more precise estimates.

The application of a multiplex technique to detect antibody responses to SARS-CoV-2 has further advantages including the assessment of additional SARS-CoV-2 proteins that might not be relevant for classification of sero-positivity but are potentially associated with disease course and/or progression, hence our reference to “serolomics,” a term that has been initially coined for Human Papillomavirus multiplex serology [22]. We here performed an explorative analysis and described the frequency of individuals with sero-positivity to multiple SARS-CoV-2 proteins other than the nucleocapsid or spike proteins, including non-structural proteins and other ORFs. Overall, *n* = 10 of hospitalized patients and none of the non-hospitalized patients were sero-positive to ≥2 SARS-CoV-2 proteins other than proteins N and S or their sub-fragments. Although sero-positivity to ≥ 2 SARS-CoV-2 proteins other than proteins N and S or their sub-fragments occurred only among a proportion of hospitalized patients, the majority of them had an overall more favorable course of disease including less invasive need for oxygenation (*n* = 9). Moreover, none of the patients with antibody responses to multiple additional SARS-CoV-2 proteins died of COVID-19. Of note, the different patient groups in this analysis differed also in age and sex (Table 4), and due to the small sample size, we were not able to appropriately control for patient characteristics in age- and sex-adjusted regression models. However, these findings raised our specific interest and should be analyzed more deeply in larger patient cohorts. A potential underlying biological mechanism for the above-described observation of a more favorable course of disease with a more pronounced antibody response to the SARS-CoV-2 proteome might be correlating B- and T-cell responses contributing to viral clearance [23].

Our study has several strengths and limitations. One major limitation is the fact that we only detected one out of 15 non-hospitalized COVID-19 patients as sero-positive. However, it should be noted that the non-hospitalized patients included in our study were all sampled within two weeks after symptom onset. In concordance with the literature [24], we observed that among hospitalized patients only after 14 days post symptom onset maximum sensitivity of the assay can be achieved. Moreover, non-hospitalized patients were of younger age and had a higher proportion of females than our control population, which might have affected the level of antibody response. A larger pre-pandemic control sample set with a wider age distribution is needed to address this question. The sensitivity among SARS-CoV-2-infected individuals experiencing mild or no symptoms also in relation to the time-point of infection needs to be further elucidated, especially with respect to future applications of the assay in large population-based cohorts. A major strength of our newly developed SARS-CoV-2 serological assay is its multiplex approach, allowing for the assessment of up to 100 antigens in parallel. It does not only permit inclusion of multiple SARS-CoV-2 proteins but also autoantigens or antigens from pathogens that might induce antibody responses due to (re-)activation or co-infection during the course of COVID-19 disease, as it was previously described for Herpesviruses [25,26,27]. We furthermore demonstrated the possibility to quantify longitudinally the humoral anti-SARS-CoV-2 responses for different Ig isotypes. In accordance with the literature (e.g., (Becker et al., 2020; Dobaño et al., 2020), we observed concomitant presence of multiple antibody isotypes in our patient cohort, especially the development of IgG antibodies at the same time as IgM. Due to the heterogeneity of the individual patient series in this study, it is, however, challenging to draw firm conclusions about isotype-specific antibody kinetics, and their use in clinical decision-making. Analysis of samples from larger cohorts of COVID-19 patients with long-term follow-up will provide information on the natural development and stability of anti-SARS-CoV-2 responses for all Ig isotype classes. In the future, it will be important to compare our assay with SARS-CoV-2- or pseudotype-based neutralization assays to correlate the levels of direct binding antibodies with neutralization capacity. Similarly important, the correlation between humoral and cellular immune responses will allow drawing a more comprehensive picture of viral clearance. The appearance of viral variants less than 1 year after onset of the pandemic is of concern, and it remains to be seen whether our assay is able to detect antibodies against variants of the spike protein, even though this appears likely due to antibody cross-reactivity, given that most viral variants are based on single amino acid changes. These topics, as well as the distinction between antibody patterns elicited by natural infection versus vaccination, are subject of ongoing studies.

In conclusion, we here present the development of a multiplex serology assay that covers a substantial proportion of the SARS-CoV-2 proteome. Considering the achieved high sensitivity and specificity of the assay and its high-throughput applicability in large sero-epidemiological studies, this assay might be suitable for efficient analysis of SARS-CoV-2 epidemiology in population-based studies. The option of hypothesis-driven inclusion of additional antigens into the panel as well as separate detection of different Ig isotypes will furthermore allow the assessment of additional research questions.

## Figures and Tables

**Table 1:** Selected proteins of SARS-CoV-2 and additional Coronaviruses for multiplex serology.

Antigen	Accession No.	Selected AA	Expression System	N-Terminal Tag	Cut-Off [MFI] ^b^
**SARS-CoV-2 (NC_045512.2)**					
NSP1	YP_009725297.1	1–180	*E. coli* BL21	GST	1107
NSP2	YP_009725298.1	1–638	*E. coli* BL21	GST	1391
NSP5	YP_009725301.1	1–306	*E. coli* BL21	GST	1044
NSP7	YP_009725303.1	1–83	*E. coli* BL21	GST	1719
NSP8	YP_009725304.1	1–198	*E. coli* BL21	GST	849
NSP9	YP_009725305.1	1–113	*E. coli* BL21	GST	605
NSP10	YP_009725306.1	1–139	*E. coli* BL21	GST	147
NSP12	YP_009725307.1	1–932	*E. coli* BL21	GST	nd
NSP13	YP_009725308.1	1–601	*E. coli* BL21	GST	nd
NSP14	YP_009725309.1	1–527	*E. coli* BL21	GST	106
NSP15	YP_009725310.1	1–346	*E. coli* BL21	GST	135
NSP16	YP_009725311.1	1–298	*E. coli* BL21	GST	387
S1	YP_009724390.1	1–685	*E. coli* BL21	GST	nd
S1 ^a^	YP_009724390.1	16–685	HEK293	His	1000
S1-RBD	YP_009724390.1	319–541	*E. coli* BL21	GST	nd
S1-RBD	YP_009724390.1	1–14, 319–541	HEK293	His	626
S2	YP_009724390.1	686–1275	*E. coli* BL21	GST	798
S2′	YP_009724390.1	817–1275	*E. coli* BL21	GST	1221
ORF3a	YP_009724391.1	1–275	*E. coli* BL21	GST	287
Protein E (ORF4)	YP_009724392.1	1–75	*E. coli* BL21	GST	122
Protein M (ORF5)	YP_009724393.1	1–222	*E. coli* BL21	GST	100
ORF6	YP_009724394.1	1–61	*E. coli* BL21	GST	100
ORF7a	YP_009724395.1	1–121	*E. coli* BL21	GST	100
ORF7b	YP_009725318.1	1–43	*E. coli* BL21	GST	100
ORF8	YP_009724396.1	1–121	*E. coli* BL21	GST	100
Protein N (ORF 9)	YP_009724397.2	1–419	*E. coli* BL21	GST	3133
N-EP3	YP_009724397.2	354–400	*E. coli* BL21	GST	1677
ORF10	YP_009725255.1	1–38	*E. coli* BL21	GST	100
**SARS-CoV (NC_004718.3)**					
Protein N	NP_828858.1	1–422	*E. coli* BL21	GST	nd
**HCoV 229E (NC_002645.1)**					
Protein N	NP_073556.1	1–389	*E. coli* BL21	GST	nd
**HCoV NL63 (NC_005831.2)**					
Protein N	YP_003771.1	1–377	*E. coli* BL21	GST	nd
**HCoV HKU1 (NC_006577.2)**					
Protein N	YP_173242.1	1–441	*E. coli* BL21	GST	nd
**HCoV OC43 (AY585228)**					
Protein N	AAT84358.1	1–448	*E. coli* BL21	GST	nd

AA, amino acid; nd, not determined; ^a^ purchased from Sino Biological (Eschborn, Germany); ^b^ Mean plus three times the standard deviation in *n* = 85 pre-pandemic (SARS-CoV-2 negative) controls for IgM/IgA/IgG antibody response; the technical minimum cut-off was 100 MFI for all antigens except S1 expressed in HEK293 cells (1000 MFI).

**Table 2:** Sero-prevalence of SARS-CoV-2 proteins in pre-pandemic controls, hospitalized, and non-hospitalized COVID-19 patients.

Antigen	N (%) Sero-Positive			
	Controls (*n* = 85)	Patients, Non-Hospitalized (*n* = 15) ^a,b^	Hospitalized Patients, ≤14 Days Post Symptom Onset (*n* = 23) ^b^	Hospitalized Patients, >14 Days Post Symptom Onset (*n* = 25) ^b^
Protein N	1 (1)	1 (7)	13 (57)	25 (100)
N-EP3	3 (4)	0 (0)	9 (39)	20 (80)
S1	0 (0)	0 (0)	11 (48)	24 (96)
S1-RBD	1 (1)	3 (20)	13 (57)	25 (100)
S2	1 (1)	1 (7)	10 (43)	20 (80)
S2′	1 (1)	0 (0)	9 (39)	19 (76)
Protein N + S1-RBD	0 (0)	1 (7)	11 (48)	25 (100)
NSP1	2 (2)	1 (7)	1 (4)	1 (4)
NSP2	2 (2)	0 (0)	2 (9)	1 (4)
NSP5	1 (1)	0 (0)	0 (0)	0 (0)
NSP7	1 (1)	0 (0)	0 (0)	1 (4)
NSP8	2 (2)	0 (0)	0 (0)	0 (0)
NSP9	1 (1)	0 (0)	2 (9)	0 (0)
NSP10	2 (2)	0 (0)	4 (17)	4 (16)
NSP14	2 (2)	0 (0)	0 (0)	4 (16)
NSP15	4 (5)	1 (7)	1 (4)	0 (0)
NSP16	1 (1)	0 (0)	2 (9)	0 (0)
ORF3a	2 (2)	0 (0)	2 (9)	3 (12)
ORF4	2 (2)	1 (7)	1 (4)	0 (0)
ORF5	0 (0)	0 (0)	4 (17)	2 (8)
ORF6	0 (0)	0 (0)	2 (9)	2 (8)
ORF7a	1 (1)	0 (0)	0 (0)	0 (0)
ORF7b	0 (0)	0 (0)	1 (4)	0 (0)
ORF8	1 (1)	0 (0)	1 (4)	1 (4)
ORF10	0 (0)	0 (0)	1 (4)	1 (4)

^a^ All sampled within 2 weeks after symptom onset; ^b^ one sample per individual drawn at the latest time point after symptom onset. All percentages were rounded.

**Table 3:** Comparison of SARS-CoV-2 sero-positivity in multiplex serology with commercially available ELISA and microscopy-based immunofluorescence detection of antibodies to SARS-CoV-2.

		ELISA (IgG) *			Microscopy-Based Immunofluorescence (IgG)		
	N (%)	Negative	Positive	Total	Negative	Positive	Total
**SARS-CoV-2 multiplex serology, Protein N + S1-RBD**	**Negative**	53 (44)	0 (0)	53 (44)	10 (27)	1 (3)	11 (30)
	**Positive**	20 (16)	49 (40)	69 (56)	0 (0)	26 (70)	26 (70)
	**Total**	73 (60)	49 (40)	122 (100)	10 (27)	27 (73)	37 (100)

* Euroimmun (Lübeck, Germany). All percentages were rounded.

**Table 4:** Age, sex, and sero-positivity to SARS-CoV-2 proteins other than protein N and S and their sub-domains and severity of disease in studied COVID-19 patients.

	Hospitalized		Oxygenation ^b^		Type of Oxygenation ^c^		Death ^b^	
	No (*n* = 15)	Yes (*n* = 48)	No (*n* = 5)	Yes (*n* = 43)	Nasal Cannula (*n* = 23)	HFNO/Invasive (*n* = 20)	No (*n* = 41)	Yes (*n* = 7)
Age (years), median (range)	54 (27–70)	62 (23–85)	50 (23–61)	63 (36–85)	55 (36–75)	69 (42–85)	59 (23–79)	78 (72–85)
Sex, *n* (%) female	9 (60%)	16 (33%)	4 (80%)	12 (28%)	8 (35%)	4 (20%)	15 (36%)	1 (14%)
Number of sero-positive proteins ^a^, *n* (%)								
0	12 (80)	22 (46)	3 (60)	19 (44)	8 (35)	11 (55)	17 (41)	5 (71)
1	3 (20)	16 (34)	1 (20)	15 (35)	7 (30)	8 (40)	14 (34)	2 (29)
2	0 (0)	5 (10)	0 (0)	5 (12)	4 (17)	1 (5)	5 (12)	0 (0)
3	0 (0)	4 (8)	0 (0)	4 (9)	4 (17)	0 (0)	4 (10)	0 (0)
4	0 (0)	0 (0)	0 (0)	0 (0)	0 (0)	0 (0)	0 (0)	0 (0)
5	0 (0)	1 (2)	1 (20)	0 (0)	0 (0)	0 (0)	1 (2)	0 (0)

HFNO, High-Flow Nasal Oxygen; ^a^ among all proteins except N, N-EP3, S1, S1-RBD, S2, and S2′; ^b^ among hospitalized patients; ^c^ among those patients that needed oxygenation. All percentages were rounded.

## Data Availability

The data presented in this study are available on request from the corresponding author. The data are not publicly available due to data protection regulations.

## Supplementary Materials

The following are available online at https://www.mdpi.com/article/10.3390/v13050749/s1, Figure S1: Antibody responses to SARS-CoV-2 proteins S1 and S1-RBD in *n* = 174 serum samples of Covid-19 patients and *n* = 85 serum samples of pre-pandemic controls, Figure S2: Correlation of antibody responses to protein N of SARS-CoV-2 to those of other CoVs. Antibody responses (MFI) to protein N of SARS-CoV-2 are compared to those of SARS-CoV-1 and ccCoV, Figure S3 a: Antibody responses (IgM/IgA/IgG) to SARS-CoV-2 non-structural proteins in longitudinal samples of *n* = 32 hospitalized Covid-19 patients, Figure S3 b: Antibody responses to SARS-CoV-2 ORF proteins in longitudinal samples of *n* = 32 hospitalized Covid-19 patients, Figure S4: Antibody response of different immunoglobulin isotypes (M, A, and G) to SARS-CoV-2 proteins N and S1-RBD in the 23 Covid-19 patients with at least 3 longitudinal samples.
