# Supplementary material for: From Multiplex Serology to Serolomics—A Novel Approach to the Antibody Response against the SARS-CoV-2 Proteome
Source: Viruses. 2021 Apr 24;13(5):749. doi: 10.3390/v13050749 (PMC8147094; doi:10.3390/v13050749)
Supplement: Supplementary file 1 [file viruses-13-00749-s001.zip › viruses-1151347-SI.pdf]

viruses

MDPI

Supplementary Materials

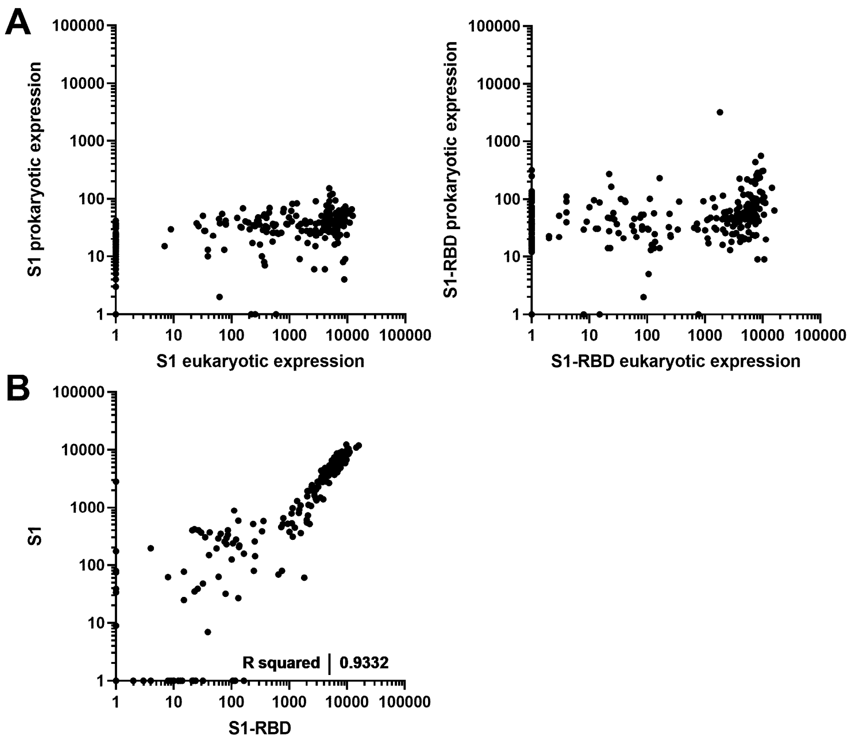

Figure S1. Antibody responses to SARS-CoV-2 proteins S1 and S1-RBD in *n* =174 serum samples of Covid-19 patients and *n* =85 serum samples of pre-pandemic controls. A) Comparison of antibody responses (MFI) to S1 and S1-RBD proteins expressed in HEK293 (eukaryotic expression) to those expressed in *E. coli* BL21 (prokaryotic expression); B) Correlation of antibody responses (MFI) to eukaryotically expressed S1 and S1-RBD; the Pearson correlation coefficient (R squared) is given as a measure for correlation.

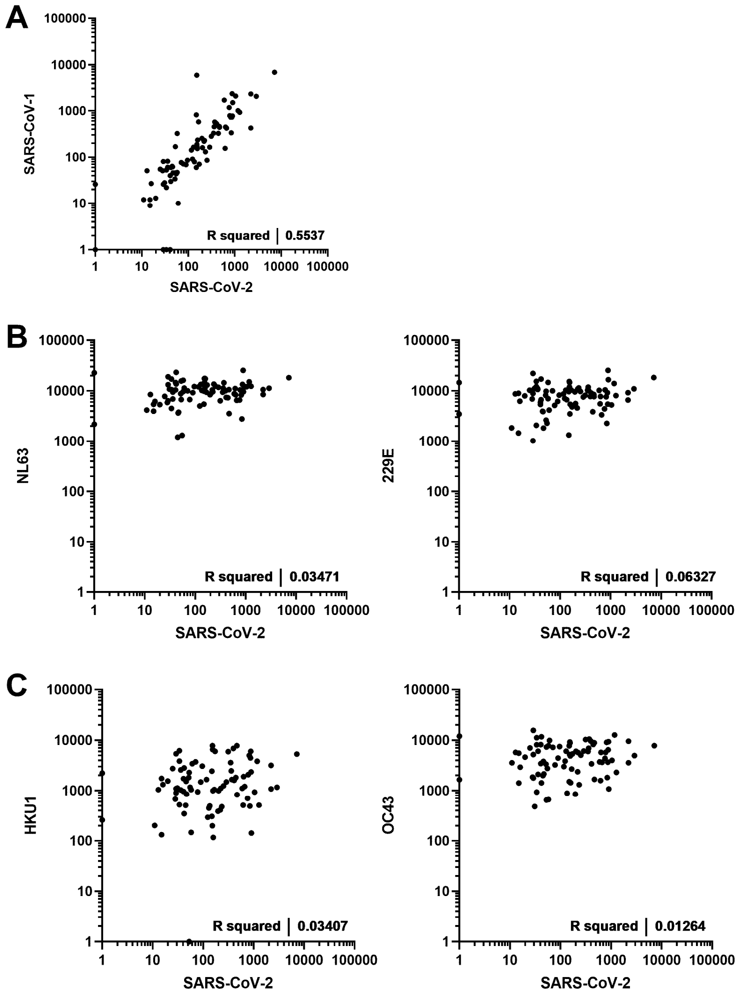

Figure S2. Correlation of antibody responses to protein N of SARS-CoV-2 to those of other CoVs. Antibody responses (MFI) to protein N of SARS-CoV-2 are compared to those of SARS-CoV-1 and ccCoVs: A) SARS-CoV-1, B) α-CoVs NL63 and 229E, and c) β-CoVs OC43 and HKU1. Blood samples were drawn prior to the Covid-19 pandemic (n =85). The Pearson correlation coefficient (R squared) is given as a measure for correlation.

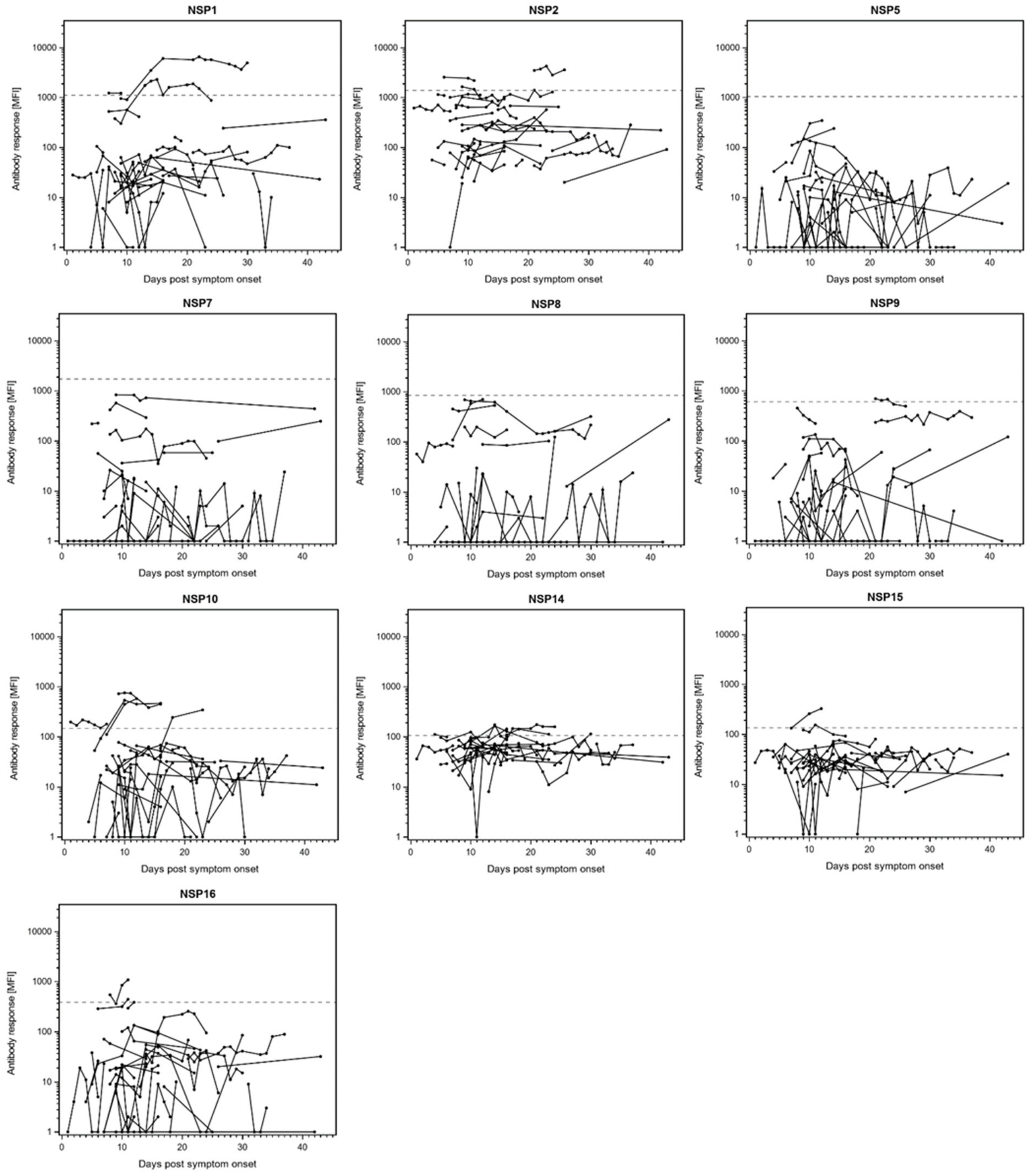

Figure S3. a: Antibody responses (IgM/IgA/IgG) to SARS-CoV-2 non-structural proteins in longitudinal samples of *n* =32 hospitalized Covid-19 patients. The antibody response (MFI) is plotted against days post symptom onset. The dashed line indicates the antigen-specific cut-off defined as mean plus three times standard deviation in *n* =85 pre-pandemic controls.

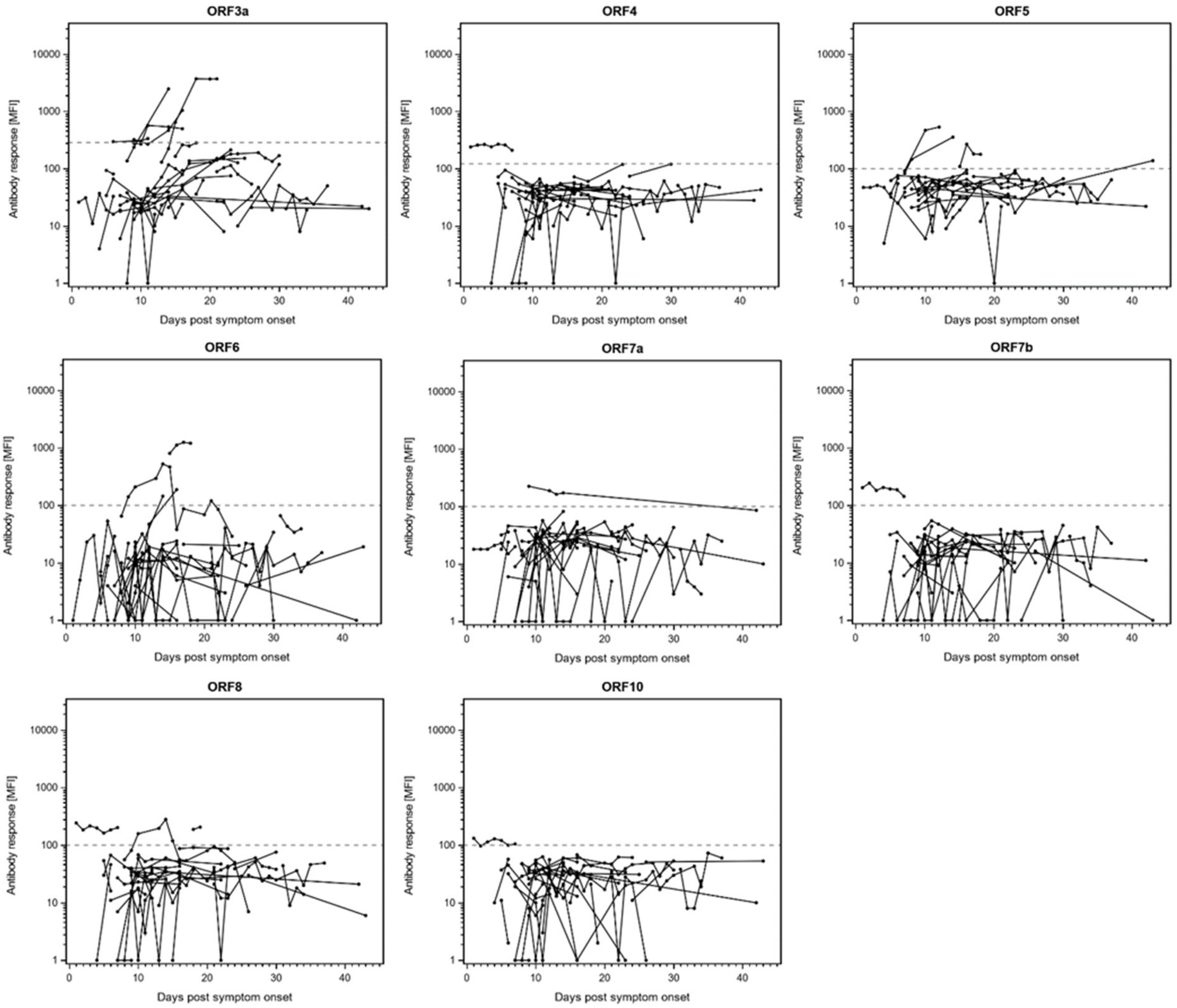

Figure S3. b: Antibody responses to SARS-CoV-2 ORF proteins in longitudinal samples of *n* =32 hospitalized Covid-19 patients. The antibody response (MFI) is plotted against days post symptom onset. The dashed line indicates the antigen-specific cut-off defined as mean plus three times standard deviation in *n* =85 pre-pandemic controls.

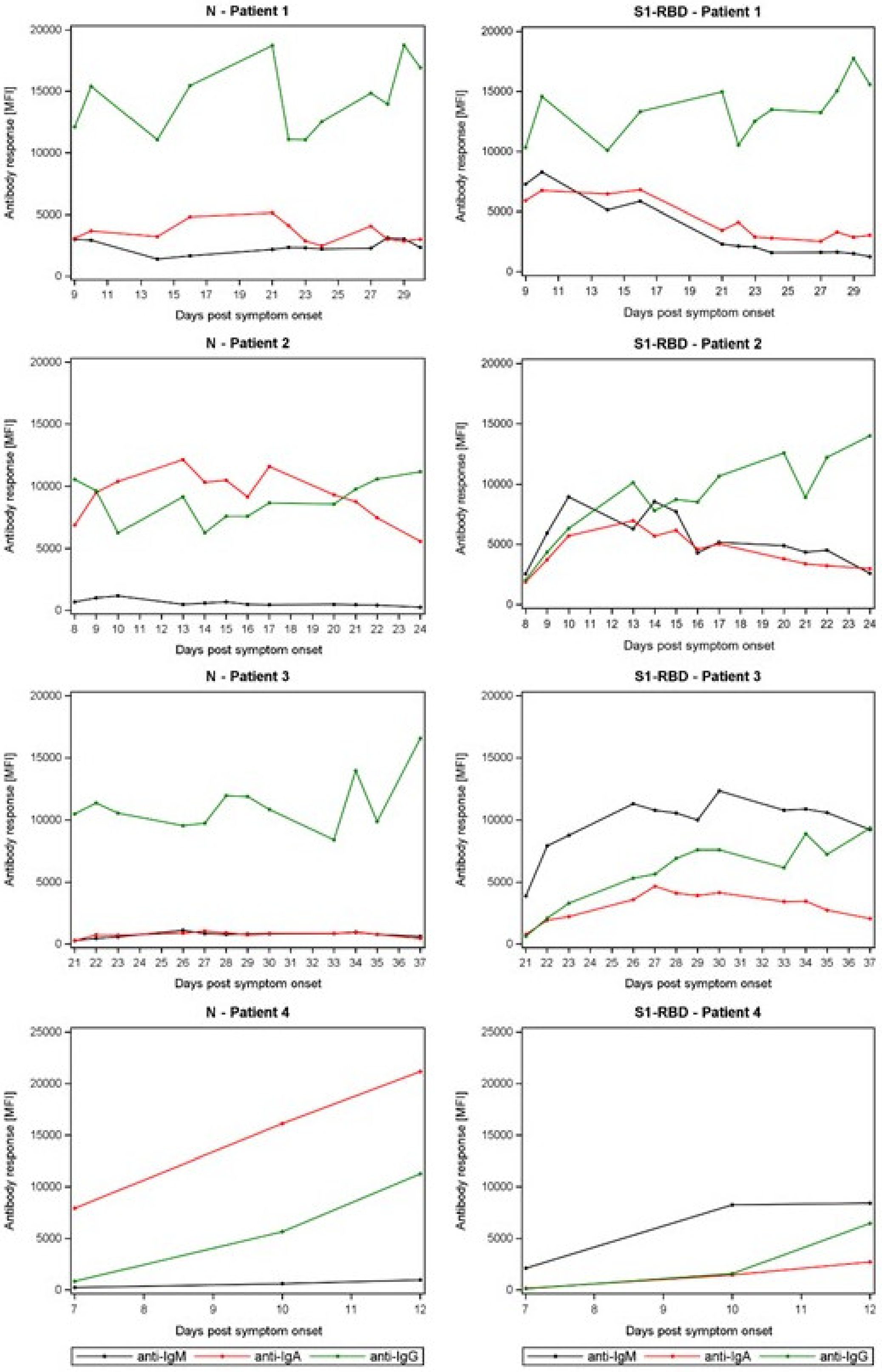
N - Patient 1
S1-RBD - Patient 1
N - Patient 2
S1-RBD - Patient 2
N - Patient 3
S1-RBD - Patient 3
N - Patient 4
S1-RBD - Patient 4
Antibody response [MFI]
Days post symptom onset
anti-IgM
anti-IgA
anti-IgG

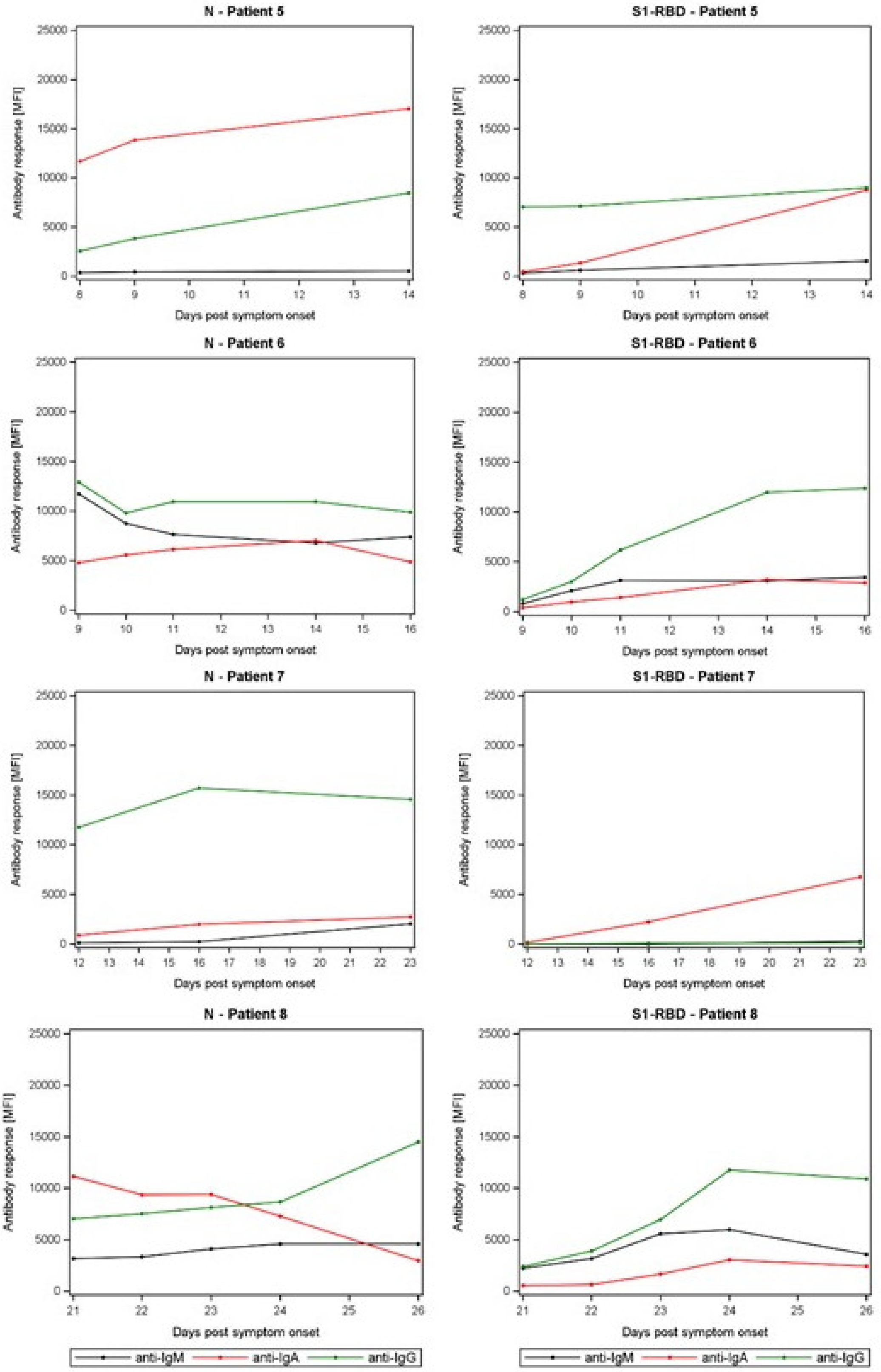
N - Patient 5
S1-RBD - Patient 5
N - Patient 6
S1-RBD - Patient 6
N - Patient 7
S1-RBD - Patient 7
N - Patient 8
S1-RBD - Patient 8
Antibody response [MFI]
Days post symptom onset
anti-IgM
anti-IgA
anti-IgG

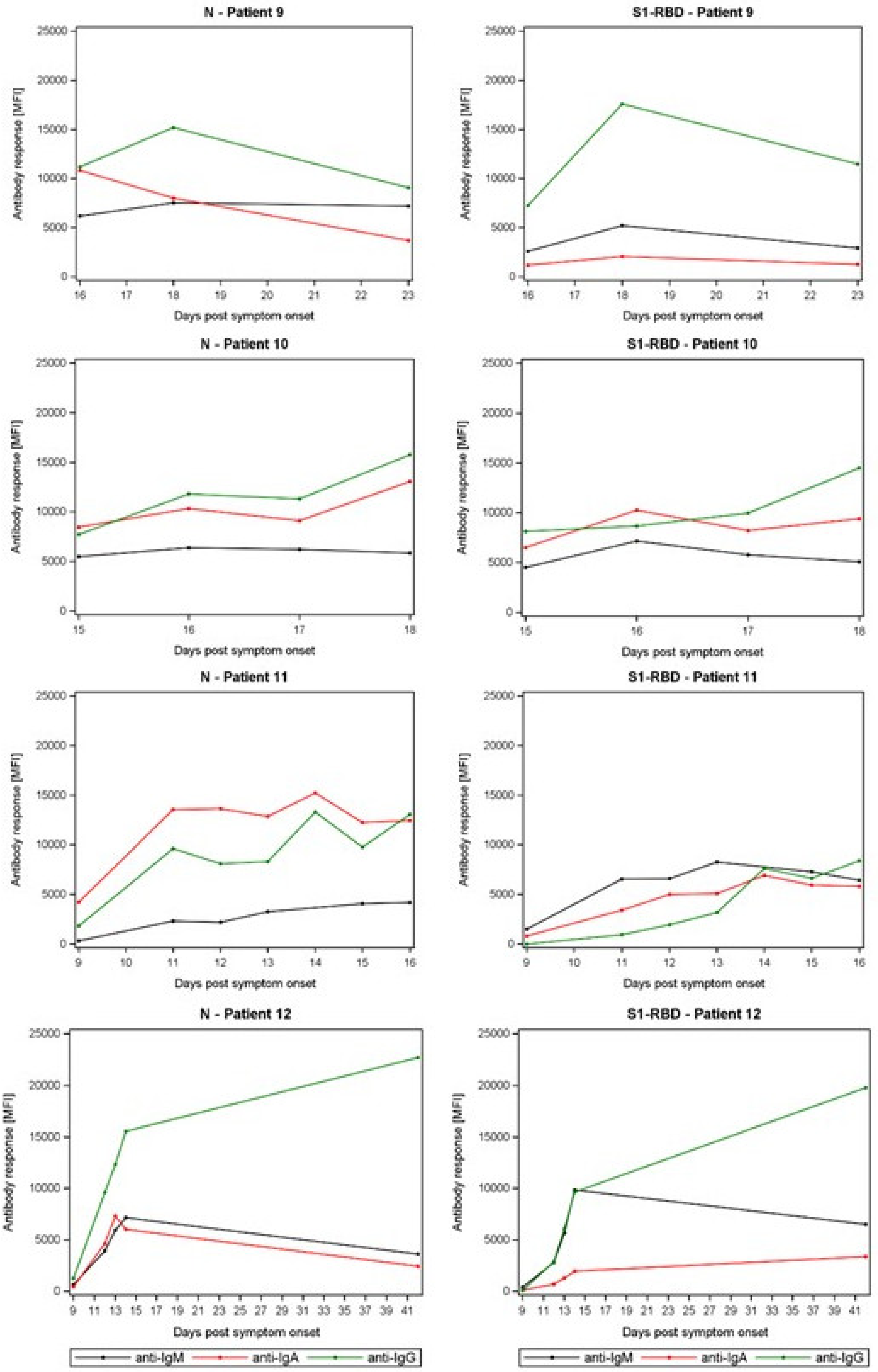
N - Patient 9
S1-RBD - Patient 9
N - Patient 10
S1-RBD - Patient 10
N - Patient 11
S1-RBD - Patient 11
N - Patient 12
S1-RBD - Patient 12
Antibody response [MFI]
Days post symptom onset
anti-IgM
anti-IgA
anti-IgG

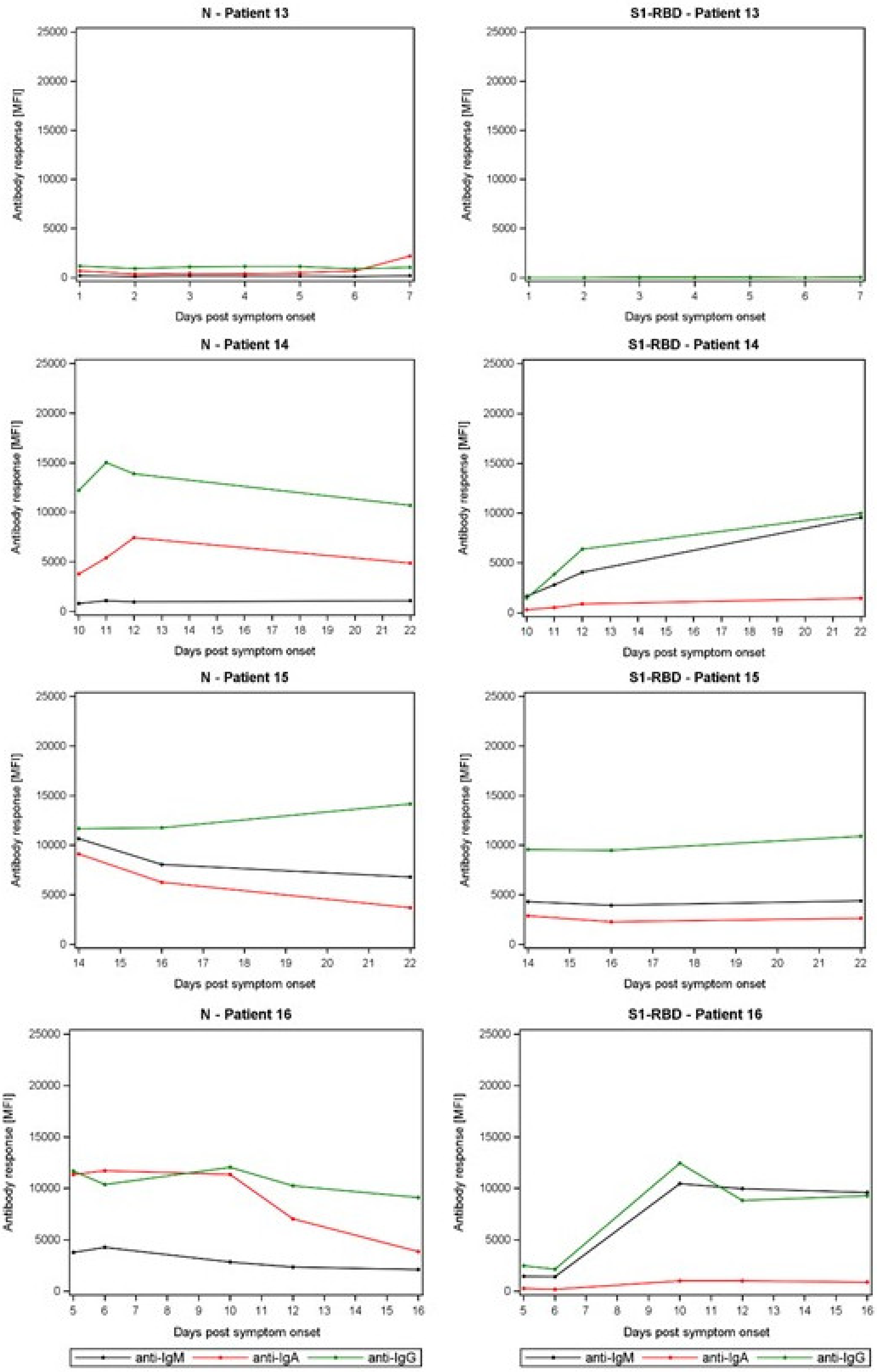
N - Patient 13
S1-RBD - Patient 13
N - Patient 14
S1-RBD - Patient 14
N - Patient 15
S1-RBD - Patient 15
N - Patient 16
S1-RBD - Patient 16
Antibody response [MFI]
Days post symptom onset
anti-IgM
anti-IgA
anti-IgG

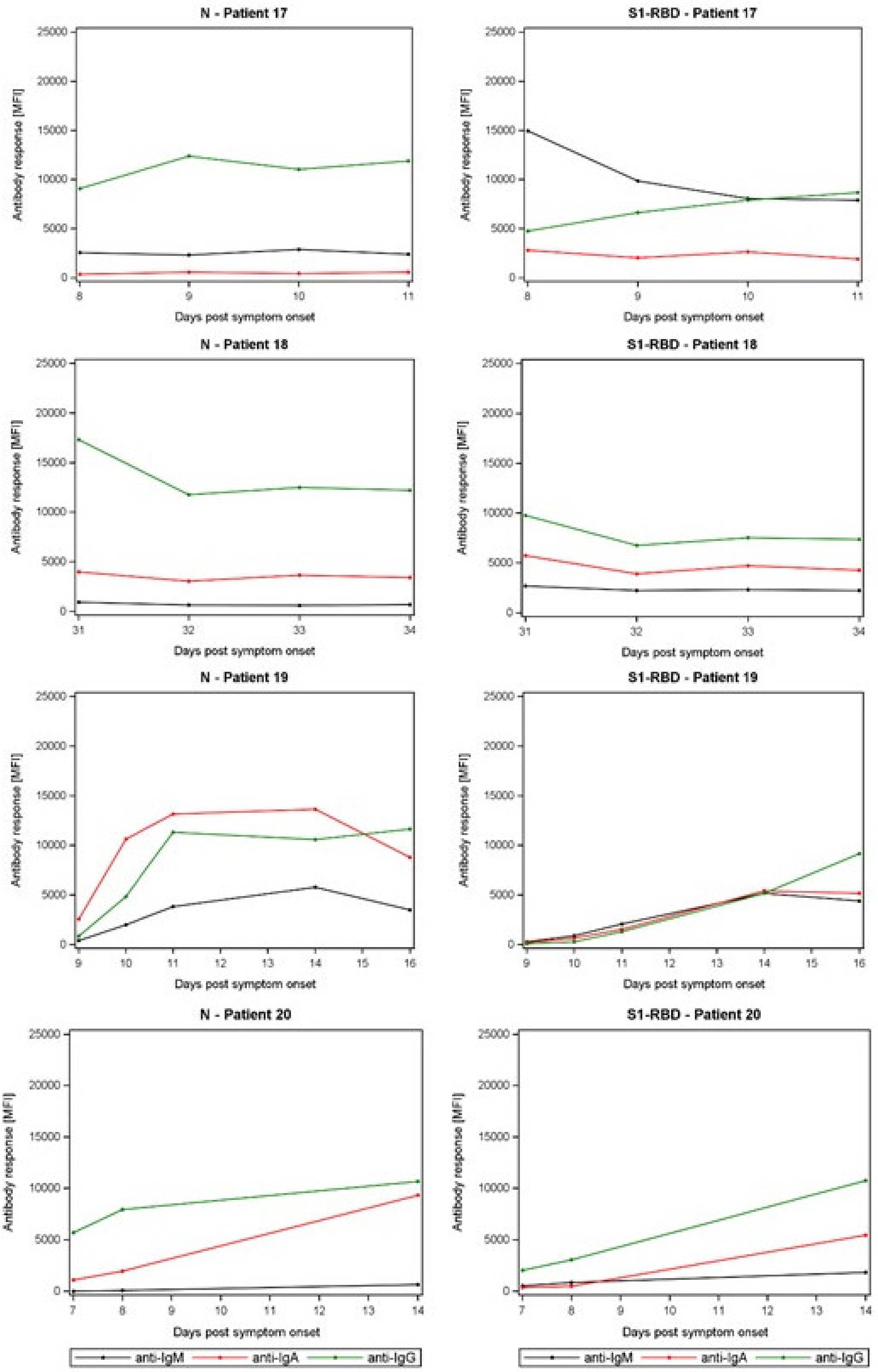
N - Patient 17
S1-RBD - Patient 17
N - Patient 18
S1-RBD - Patient 18
N - Patient 19
S1-RBD - Patient 19
N - Patient 20
S1-RBD - Patient 20
Antibody response [MFI]
Days post symptom onset
anti-IgM
anti-IgA
anti-IgG

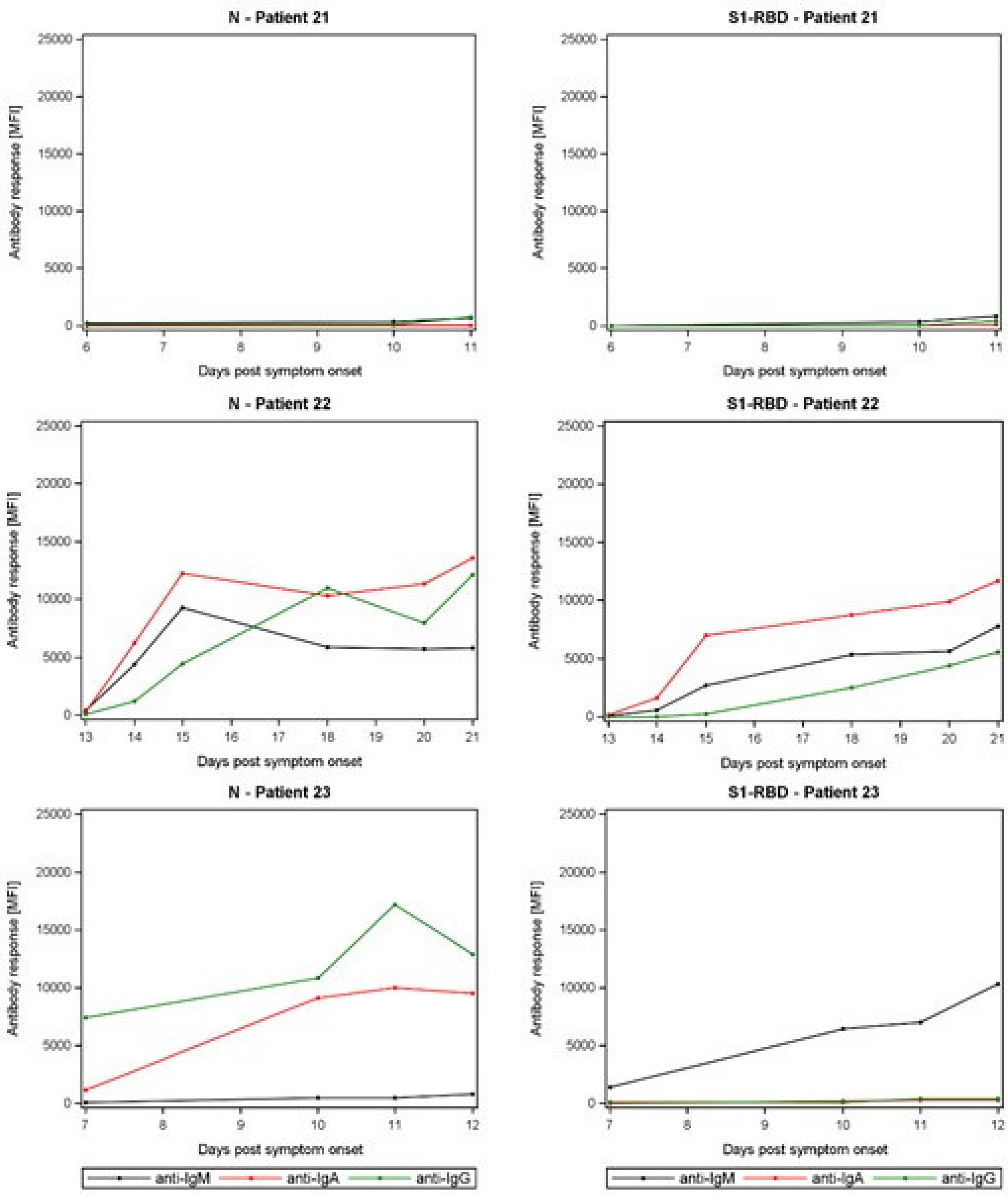

Figure S4. Antibody response of different immunoglobulin isotypes (M, A, and G) to SARS-CoV-2 proteins N and S1-RBD in the 23 Covid-19 patients with at least 3 longitudinal samples. The IgM, IgA, and IgG antibody response (MFI) is plotted against days post symptom onset.
